# Supplementary material for: Adaptive memory reservation strategy for heavy workloads in the Spark environment
Source: PeerJ Comput Sci. 2024 Nov 13;10:e2460. doi: 10.7717/peerj-cs.2460 (PMC11639302; doi:10.7717/peerj-cs.2460)
Supplement: Supplemental Information 2 [file peerj-cs-10-2460-s002.docx]

The datasets need to be generated on local servers under identical environmental conditions, producing various types and sizes of datasets for multiple tests. When comparing different strategies, it is crucial to use the same dataset for testing and conduct multiple comparisons using different datasets.
