# Supplementary material for: Adaptive memory reservation strategy for heavy workloads in the Spark environment
Source: PeerJ Comput Sci. 2024 Nov 13;10:e2460. doi: 10.7717/peerj-cs.2460 (PMC11639302; doi:10.7717/peerj-cs.2460)
Supplement: Supplemental Information 3 [file peerj-cs-10-2460-s003.zip › scala-2.12.11/doc/License.rtf]

Scala is licensed under the Apache License Version 2.0.Scala LicenseCopyright (c) 2002-2020 EPFLCopyright (c) 2011-2020 Lightbend, Inc.All rights reserved.Licensed under the Apache License, Version 2.0 (the "License"); you may not use this file except in compliance with the License. You may obtain a copy of the License at http://www.apache.org/licenses/LICENSE-2.0.Unless required by applicable law or agreed to in writing, software distributed under the License is distributed on an "AS IS" BASIS, WITHOUT WARRANTIES OR CONDITIONS OF ANY KIND, either express or implied. See the License for the specific language governing permissions and limitations under the License.Other LicensesThis software includes projects with the following licenses, which are also included in the licenses/ directory:Apache LicenseThis license is used by the following third-party libraries:	•jansiBSD LicenseThis license is used by the following third-party libraries:	•jlineBSD 3-Clause LicenseThis license is used by the following third-party libraries:	•asmMIT LicenseThis license is used by the following third-party libraries:	•jquery	•tools tooltip
