# Supplementary material for: Adaptive memory reservation strategy for heavy workloads in the Spark environment
Source: PeerJ Comput Sci. 2024 Nov 13;10:e2460. doi: 10.7717/peerj-cs.2460 (PMC11639302; doi:10.7717/peerj-cs.2460)
Supplement: Supplemental Information 3 [file peerj-cs-10-2460-s003.zip › scala-2.12.11/doc/tools/scaladoc.html]

xml version="1.1" encoding="iso-8859-1"?


scaladoc man page


scaladoc(1)

scaladoc(1)

USER COMMANDS

### NAME

`scaladoc` – Documentation generator for the Scala 2 language

### SYNOPSIS

`scaladoc` `[ <options> ] <source files>`

### PARAMETERS

`<options>`
:   Command line options. See **OPTIONS** below.

`<source files>`
:   One or more source files to be compiled (such as `MyClass.scala`).

### DESCRIPTION

The `scaladoc` tool reads class and object definitions, written in the Scala 2 programming language, and generates their API as HTML files.

By default, the generator puts each HTML file in the same directory as its source file. You can specify a separate destination directory with `–d` (see **OPTIONS**, below).

The recognised format of comments in source is described in the online documentation

### OPTIONS

#### Standard Options

`–d <directory>`
:   Specify where to generate documentation.

`–version`
:   Print product version and exit.

`–help`
:   Print a synopsis of available options.

#### Documentation Options

`–doc-title <title>`
:   Define the overall title of the documentation, typically the name of the library being documented.

`–doc-version <version>`
:   Define the overall version number of the documentation, typically the version of the library being documented.

`–doc-source-url <url>`
:   Define a URL to be concatenated with source locations for link to source files.

`–doc-external-doc <external-doc>`
:   Define a comma-separated list of classpath\_entry\_path#doc\_URL pairs describing external dependencies.

#### Compiler Options

`–verbose`
:   Output messages about what the compiler is doing

`–deprecation`
:   Indicate whether source should be compiled with deprecation information; defaults to `off` (accepted values are: `on`, `off`, `yes` and `no`)

    Available since Scala version 2.2.1

`–classpath <path>`
:   Specify where to find user class files (on Unix-based systems a colon-separated list of paths, on Windows-based systems, a semicolon-separate list of paths). This does not override the built-in (`"boot"`) search path.

    The default class path is the current directory. Setting the `CLASSPATH` variable or using the `-classpath` command-line option overrides that default, so if you want to include the current directory in the search path, you must include `"."` in the new settings.

`–sourcepath <path>`
:   Specify where to find input source files.

`–bootclasspath <path>`
:   Override location of bootstrap class files (where to find the standard built-in classes, such as "`scala.List`").

`–extdirs <dirs>`
:   Override location of installed extensions.

`–encoding <encoding>`
:   Specify character encoding used by source files.

    The default value is platform-specific (Linux: `"UTF8"`, Windows: `"Cp1252"`). Executing the following code in the Scala interpreter will return the default value on your system:

    `scala>` `new java.io.InputStreamReader(System.in).getEncoding`

### EXIT STATUS

`scaladoc` returns a zero exit status if it succeeds at processing the specified input files. Non zero is returned in case of failure.

### AUTHORS

This version of Scaladoc was written by Gilles Dubochet with contributions by Pedro Furlanetto and Johannes Rudolph. It is based on the original Scaladoc (Sean McDirmid, Geoffrey Washburn, Vincent Cremet and Stéphane Micheloud), on vScaladoc (David Bernard), as well as on an unreleased version of Scaladoc 2 (Manohar Jonnalagedda).

### SEE ALSO

**fsc**(1), **scala**(1), **scalac**(1), **scalap**(1)

version 2.0

scaladoc(1)

June 2010
