# Supplementary material for: Adaptive memory reservation strategy for heavy workloads in the Spark environment
Source: PeerJ Comput Sci. 2024 Nov 13;10:e2460. doi: 10.7717/peerj-cs.2460 (PMC11639302; doi:10.7717/peerj-cs.2460)
Supplement: Supplemental Information 3 [file peerj-cs-10-2460-s003.zip › scala-2.12.11/doc/tools/scalap.html]

xml version="1.1" encoding="iso-8859-1"?


scalap man page


scalap(1)

scalap(1)

USER COMMANDS

### NAME

`scalap` – Scala class file decoder for the Scala 2 language

### SYNOPSIS

`scalap` `[ <options> ] <class name>`

### PARAMETERS

`<options>`
:   Command line options. See **OPTIONS** below.

`<class name>`
:   Full-qualified name of a class to be decoded (such as `hello.HelloWorld`).

### DESCRIPTION

The `scalap` tool reads a class file generated by theScala compiler, and displays object and class definitions.

By default, `scalap` looks for the given class file in the current directory. You can specify a separate classpath with `–classpath` (see **OPTIONS**, below).

### OPTIONS

The decoder has a set of standard options that are supported on the current development environment and will be supported in future releases.

#### Standard Options

`–help`
:   Display this usage message.

`–private`
:   Print private definitions.

`–verbose`
:   Print out additional information.

`–version`
:   Print product version and exit.

`–cp` | `–classpath`
:   Specify where to find user class files.

### EXAMPLES

Display definitions for a generated class file
:   `scalap` `hello.HelloWorld`

### EXIT STATUS

`scalap` returns a zero exit status if it succeeds to process the specified input files. Non zero is returned in case of failure.

### AUTHOR

Written by Ilya Sergey.

### REPORTING BUGS

Report bugs to `https://github.com/scala/bug/issues`.

### COPYRIGHT

This is open-source software, available to you under the Apache License 2.0. See accompanying "copyright" or "LICENSE" file for copying conditions. There is NO warranty; not even for MERCHANTABILITY or FITNESS FOR A PARTICULAR PURPOSE.

### SEE ALSO

**fsc**(1), **scala**(1), **scalac**(1), **scaladoc**(1)

version 1.0

scalap(1)

June 2006
