# Supplementary material for: Adaptive memory reservation strategy for heavy workloads in the Spark environment
Source: PeerJ Comput Sci. 2024 Nov 13;10:e2460. doi: 10.7717/peerj-cs.2460 (PMC11639302; doi:10.7717/peerj-cs.2460)
Supplement: Supplemental Information 3 [file peerj-cs-10-2460-s003.zip › scala-2.12.11/doc/tools/scala.html]

xml version="1.1" encoding="iso-8859-1"?


scala man page


scala(1)

scala(1)

USER COMMANDS

### NAME

`scala` – Run code in the Scala 2 language

### SYNOPSIS

`scala` `[ <option> ]... [ <torun> <argument>... ]`

### PARAMETERS

`<compiler-option>`
:   Any scalac option. See **scalac**(1).

`–howtorun:<how>`
:   How to execute <*torun*>, if it is present. Options for <*how*> are `guess` (the default), `script`, `jar`, and `object`.

`–i <file>`
:   Requests that a file be pre-loaded. It is only meaningful for interactive shells.

`–e <string>`
:   Requests that its argument be executed as Scala code.

`–savecompiled`
:   Save this compiled version of scripts in order to speed up later executions of the same script. When running a script, save the compiled version in a file with the same name as the script but with an extension of `.jar`. On subsequent runs of the same script, the pre-compiled `.jar` file will be used if it is newer than the script file.

`–nocompdaemon`
:   Do not use the `fsc` offline compiler.

`–nc`
:   Same as `-nocompdaemon`.

`–Dproperty=value`
:   Set a Java system property. If no value is specified, then the property is set to the empty string.

`<torun>`
:   A top-level object or a script file to run.

`<argument>`
:   An arguments to pass to <*torun*>.

### DESCRIPTION

The `scala` utility runs Scala code using a Java runtime environment. The Scala code to run is specified in one of three ways:

1. With no arguments specified, a Scala shell starts and reads commands interactively.- With `-howtorun:object` specified, the fully qualified name of a top-level Scala object may be specified. The object should previously have been compiled using **scalac**(1).- With `-howtorun:script` specified, a file containing Scala code may be specified.

If `-howtorun:` is left as the default (`guess`), then the `scala` command will check whether a file of the specified name exists. If it does, then it will treat it as a script file; if it does not, then it will treat it as the name of an object.

In all three cases, arbitrary scalac options may be specified. The most common option is to specify a classpath with `-classpath`, but see the **scalac**(1) page for full details.

If an object is specified to run, then that object must be a top-level Scala object with the specified name. The object must define a method **main** with the following signature:

> `def main(args: Array[String]): Unit`

The method must return a **Unit** value, and it must accept a **String** array as a parameter. All arguments specified on the command line will be passed as arguments to the **main** method.

If a script file is specified to run, then the file is read and all Scala statements and declarations in the file are processed in order. Any arguments specified will be available via the `args`variable.

Script files may have an optional header that is ignored if present. There are two ways to format the header: either beginning with `#!` and ending with `!#`, or beginning with `::#!` and ending with `::!#`.

Such a header must have each header boundary start at the beginning of a line. Headers can be used to make stand-alone script files, as shown in the examples below.

When running a script or using `-e`, an already running compilation daemon (fsc) is used, or a new one started on demand. The `-nocompdaemon` or `-nc` option can be used to prevent this.

If no -classpath option is specified, then `scala` will add ".", the current directory, to the end of the classpath.

### OPTIONS

If any compiler options are specified, they must be first in the command line and must be followed by a bare hyphen ("-") character. If no arguments are specified after the optional compiler arguments, then an interactive Scala shell is started. Otherwise, either a script file is run, or a pre-compiled Scala object is run. It is possible to distinguish the last two cases by using an explicit `-object` or `-script` flag, but usually the program can guess correctly.

### ENVIRONMENT

`JAVACMD`
:   Specify the `java` command to be used for running the Scala code. Arguments may be specified as part of the environment variable; spaces, quotation marks, etc., will be passed directly to the shell for expansion.

`JAVA_HOME`
:   Specify JDK/JRE home directory. This directory is used to locate the `java` command unless `JAVACMD` variable set.

`JAVA_OPTS`
:   Specify the options to be passed to the `java` command defined by `JAVACMD`.

    With Java 1.5 (or newer) one may for example configure the memory usage of the JVM as follows: `JAVA_OPTS="-Xmx512M -Xms16M -Xss16M"`

### EXAMPLES

Here are some examples of running Scala code:

Execute a Scala program generated in the current directory
:   `scala` `hello.HelloWorld`

Execute a Scala program generated in a user-defined directory **classes**
:   `scala` `–classpath classes hello.HelloWorld`

Execute a Scala program using a user-defined `java` command
:   `env JAVACMD``=/usr/local/bin/cacao` `scala` `–classpath classes hello.HelloWorld`

Execute a Scala program using JVM options
:   `env JAVACMD``=java` `JAVA_OPTS``="-Dmsg=hello -enableassertions"` `scala` `–classpath classes hello.HelloWorld`

Here is a complete Scala script for Unix:

```
#!/bin/sh
exec scala "$0" "$@"
!#
Console.println("Hello, world!")
args.toList foreach Console.println
```

Here is a complete Scala script for MS Windows:

```
::#!
@echo off
call scala %0 %*
goto :eof
::!#
Console.println("Hello, world!")
args.toList foreach Console.println
```

If you want to use the compilation cache to speed up multiple executions of the script, then add `-savecompiled` to the scala command:

```
#!/bin/sh
exec scala -savecompiled "$0" "$@"
!#
Console.println("Hello, world!")
args.toList foreach Console.println
```

### EXIT STATUS

The `scala` command returns a zero exit status if it succeeds. Non zero is returned in case of any error. If a script or top-level object is executed and returns a value, then that return value is passed on to `scala`.

### AUTHOR

Written by Martin Odersky and other members of the Scala team.

### REPORTING BUGS

Report bugs to `https://github.com/scala/bug/issues`.

### COPYRIGHT

This is open-source software, available to you under the Apache License 2.0. See accompanying "copyright" or "LICENSE" file for copying conditions. There is NO warranty; not even for MERCHANTABILITY or FITNESS FOR A PARTICULAR PURPOSE.

### SEE ALSO

**fsc**(1), **scalac**(1), **scaladoc**(1), **scalap**(1)

version 0.5

scala(1)

April 2007
