# Supplementary material for: Adaptive memory reservation strategy for heavy workloads in the Spark environment
Source: PeerJ Comput Sci. 2024 Nov 13;10:e2460. doi: 10.7717/peerj-cs.2460 (PMC11639302; doi:10.7717/peerj-cs.2460)
Supplement: Supplemental Information 3 [file peerj-cs-10-2460-s003.zip › scala-2.12.11/doc/tools/index.html]

xml version="1.1" encoding="iso-8859-1"?


Scala Development Tools


## Scala Tools and Utilities

### General

- **General Information**

  (classpath, how classes are found)

### Standard Scala Tools and Utilities

- **Basic Tools** (`fsc`,
  `scala`, `scalac`, `scaladoc`,
  `scalap`)

**NOTE** - Some tools have separate reference pages for Windows, Linux and Solaris
to accommodate minor differences in configuration and usage -- for example, the character
used to specify directory separators may be different.

---

## General Information

The following documents contain important information you will need to
know to get the most out of the SDK tools.

|  |  |
| --- | --- |
| **Setting the Classpath** | [Solaris and Linux] [Windows] |
| **How Classes are Found** | [Solaris, Linux and Windows] |

---

## Basic Tools

These tools are the foundation of the Scala SDK. They are the tools you
use to create and build applications.

| Tool Name | Brief Description | Links to Reference Pages |
| --- | --- | --- |
| fsc | The fast Scala compiler. | [Solaris, Linux and Windows] |
| scala | Run Scala code. | [Solaris, Linux and Windows] |
| scalac | Compile Scala code ahead of time. | [Solaris, Linux and Windows] |
| scaladoc | The API document generator. | [Solaris, Linux and Windows] |
| scalap | The Scala class file decoder. | [Solaris, Linux and Windows] |

---
