# Supplementary material for: Adaptive memory reservation strategy for heavy workloads in the Spark environment
Source: PeerJ Comput Sci. 2024 Nov 13;10:e2460. doi: 10.7717/peerj-cs.2460 (PMC11639302; doi:10.7717/peerj-cs.2460)
Supplement: Supplemental Information 3 [file peerj-cs-10-2460-s003.zip › scala-2.12.11/doc/tools/scalac.html]

xml version="1.1" encoding="iso-8859-1"?


scalac man page


scalac(1)

scalac(1)

USER COMMANDS

### NAME

`scalac` – Compiler for the Scala 2 language

### SYNOPSIS

`scalac` `[ <options> ] <source files>`

### PARAMETERS

`<options>`
:   Command line options. See **OPTIONS** below.

`<source files>`
:   One or more source files to be compiled (such as `MyClass.scala`).

### DESCRIPTION

The `scalac` tool reads class and object definitions, written in the Scala programming language, and compiles them into bytecode class files.

By default, the compiler puts each class file in the same directory as its source file. You can specify a separate destination directory with -d (see **OPTIONS**, below).

### OPTIONS

The compiler has a set of standard options that are supported on the current development environment and will be supported in future releases. An additional set of non-standard options are specific to the current virtual machine implementation and are subject to change in the future. Non-standard options begin with `-X`.

#### Standard Options

`–Dproperty=value`
:   Pass `–Dproperty=value`  directly to the runtime system.

`–J<flag>`
:   Pass `<flag>` directly to the runtime system.

`–P:<plugin:opt>`
:   Pass an option to a plugin

`–X`
:   Print a synopsis of advanced options.

`–bootclasspath <path>`
:   Override location of bootstrap class files (where to find the standard built-in classes, such as "`scala.List`").

`–classpath <path>`
:   Specify where to find user class files (on Unix-based systems a colon-separated list of paths, on Windows-based systems, a semicolon-separated list of paths). This does not override the built-in (`"boot"`) search path.

    The default class path is the current directory. Setting the `CLASSPATH` variable or using the `-classpath` command-line option overrides that default, so if you want to include the current directory in the search path, you must include `"."` in the new settings.

`–d <directory|jar>`
:   Specify where to place generated class files.

`–deprecation`
:   Emit warning and location for usages of deprecated APIs.

    Available since Scala version 2.2.1

`–encoding <encoding>`
:   Specify character encoding used by source files.

    The default value is platform-specific (Linux: `"UTF8"`, Windows: `"Cp1252"`). Executing the following code in the Scala interpreter will return the default value on your system:

    `scala>` `new java.io.InputStreamReader(System.in).getEncoding`

`–explaintypes`
:   Explain type errors in more detail.

`–extdirs <dirs>`
:   Override location of installed extensions.

`–feature`
:   Emit warning and location for usages of features that should be imported explicitly.

`–g:{none,source,line,vars,notailcalls}`
:   `"none"` generates no debugging info,

    `"source"` generates only the source file attribute,

    `"line"` generates source and line number information,

    `"vars"` generates source, line number and local variable information,

    `"notailcalls"` generates all of the above and *will not* perform tail call optimization.

`–help`
:   Print a synopsis of standard options.

`–javabootclasspath <path>`
:   Override Java boot classpath.

`–javaextdirs <path>`
:   Override Java extdirs classpath.

`–language:<feature>`
:   Enable one or more language features.

`–no-specialization`
:   Ignore `@specialize` annotations.

`–nobootcp`
:   Do not use the boot classpath for the Scala jar files.

`–nowarn`
:   Generate no warnings

`–optimise`
:   Generates faster bytecode by applying optimisations to the program.

`–print`
:   Print program with all Scala-specific features removed.

`–sourcepath <path>`
:   Specify location(s) of source files.

`–target:{jvm-1.8}`
:   `"jvm-1.8"` target JVM 1.8 (default)

`–toolcp <path>`
:   Add to the runner classpath.

`–unchecked`
:   Enable detailed unchecked (erasure) warnings

    Non variable type-arguments in type patterns are unchecked since they are eliminated by erasure

    Available since Scala version 2.3.0

`–uniqid`
:   Uniquely tag all identifiers in debugging output.

`–usejavacp`
:   Utilize the java.class.path in classpath resolution.

`–usemanifestcp`
:   Utilize the manifest in classpath resolution.

`–verbose`
:   Output messages about what the compiler is doing

`–version`
:   Print product version and exit.

`@<file>`
:   A text file containing compiler arguments (options and source files)

#### Advanced Options

`–Xcheckinit`
:   Wrap field accessors to throw an exception on uninitialized access.

`–Xdev`
:   Enable warnings for developers working on the Scala compiler

`–Xdisable-assertions`
:   Generate no assertions and assumptions

`–Xelide-below <n>`
:   Calls to `@elidable` methods are omitted if method priority is lower than argument.

`–Xexperimental`
:   Enable experimental extensions

`–Xfatal-warnings`
:   Fail the compilation if there are any warnings.

`–Xfull-lubs`
:   Retain pre 2.10 behavior of less aggressive truncation of least upper bounds.

`–Xfuture`
:   Turn on future language features.

`–Xgenerate-phase-graph <file>`
:   Generate the phase graphs (outputs .dot files) to fileX.dot.

`–Xlint`
:   Enable recommended additional warnings.

`–Xlog-free-terms`
:   Print a message when reification creates a free term.

`–Xlog-free-types`
:   Print a message when reification resorts to generating a free type.

`–Xlog-implicit-conversions`
:   Print a message whenever an implicit conversion is inserted.

`–Xlog-implicits`
:   Show more detail on why some implicits are not applicable.

`–Xlog-reflective-calls`
:   Print a message when a reflective method call is generated.

`–Xmacro-settings:<option>`
:   Custom settings for macros.

`–Xmain-class <path>`
:   Class for manifest's Main-Class entry (only useful with -d <jar>).

`–Xmax-classfile-name <n>`
:   Maximum filename length for generated classes.

`–Xmigration:<version>`
:   Warn about constructs whose behavior may have changed since<*version*>.

`–Xno-forwarders`
:   Do not generate static forwarders in mirror classes.

`–Xno-patmat-analysis`
:   Don't perform exhaustivity/unreachability analysis. Also, ignore `@switch` annotation.

`–Xno-uescape`
:   Disable handling of \u unicode escapes

`–Xnojline`
:   Do not use JLine for editing.

`–Xplugin:<paths>`
:   Load a plugin from each classpath.

`–Xplugin-disable:<plugin>`
:   Disable plugins by name.

`–Xplugin-list`
:   Print a synopsis of loaded plugins.

`–Xplugin-require:<plugin>`
:   Abort if a named plugin is not loaded.

`–Xpluginsdir <path>`
:   Path to search for plugin archives.

`–Xprint:<phases>`
:   Print out program after <*phases*> (see below).

`–Xprint-icode[:<phases>]`
:   Log internal icode to \*.icode files after<*phases*> (default: icode).

`–Xprint-pos`
:   Print tree positions, as offsets.

`–Xprint-types`
:   Print tree types (debugging option).

`–Xprompt`
:   Display a prompt after each error (debugging option).

`–Xresident`
:   Compiler stays resident, files to compile are read from standard input.

`–Xscript <object>`
:   Treat the source file as a script and wrap it in a main method.

`–Xshow-class <class>`
:   Show internal representation of class.

`–Xshow-object <object>`
:   Show internal representation of object.

`–Xshow-phases`
:   Print a synopsis of compiler phases.

`–Xsource:<version>`
:   Treat compiler input as Scala source for the specified version, see scala/bug#8126.

`–Xsource-reader <classname>`
:   Specify a custom method for reading source files.

`–Xstrict-inference`
:   Don't infer known-unsound types.

`–Xverify`
:   Verify generic signatures in generated bytecode (asm backend only).

`–Xxml:{coalescing}`
:   Configure XML parsing.

    `"coalescing"` convert PCData to Text and coalesce sibling nodes (default in 2.11).

`–Y`
:   Print a synopsis of private options.

#### Compilation Phases

`parser`
:   parse source into ASTs, perform simple desugaring

`namer`
:   resolve names, attach symbols to named trees

`packageobjects`
:   load package objects

`typer`
:   the meat and potatoes: type the trees

`patmat`
:   translate match expressions

`superaccessors`
:   add super accessors in traits and nested classes

`extmethods`
:   add extension methods for inline classes

`pickler`
:   serialize symbol tables

`refchecks`
:   reference/override checking, translate nested objects

`selectiveanf`
:   ANF pre-transform for `@cps` (CPS plugin)

`selectivecps`
:   `@cps`-driven transform of selectiveanf assignments (CPS plugin)

`uncurry`
:   uncurry, translate function values to anonymous classes

`tailcalls`
:   replace tail calls by jumps

`specialize`
:   `@specialized`-driven class and method specialization

`explicitouter`
:   this refs to outer pointers, translate patterns

`erasure`
:   erase types, add interfaces for traits

`posterasure`
:   clean up erased inline classes

`fields`
:   synthesize accessors and fields, including bitmaps for lazy vals

`lambdalift`
:   move nested functions to top level

`constructors`
:   move field definitions into constructors

`flatten`
:   eliminate inner classes

`mixin`
:   mixin composition

`cleanup`
:   platform-specific cleanups, generate reflective calls

`delambdafy`
:   remove lambdas

`icode`
:   generate portable intermediate code

`inliner`
:   optimization: do inlining

`inlineHandlers`
:   optimization: inline exception handlers

`closelim`
:   optimization: eliminate uncalled closures

`constopt`
:   optimization: optimize null and other constants

`dce`
:   optimization: eliminate dead code

`jvm`
:   generate JVM bytecode

`terminal`
:   the last phase in the compiler chain

`all`
:   matches all phases

### ENVIRONMENT

`JAVACMD`
:   Specify the `java` command to be used for running the Scala code. Arguments may be specified as part of the environment variable; spaces, quotation marks, etc., will be passed directly to the shell for expansion.

`JAVA_HOME`
:   Specify JDK/JRE home directory. This directory is used to locate the `java` command unless `JAVACMD` variable set.

`JAVA_OPTS`
:   Specify the options to be passed to the `java` command defined by `JAVACMD`.

    With Java 1.5 (or newer) one may for example configure the memory usage of the JVM as follows: `JAVA_OPTS="-Xmx512M -Xms16M -Xss16M"`

### EXAMPLES

Compile a Scala program to the current directory
:   `scalac` `HelloWorld`

Compile a Scala program to the destination directory `classes`
:   `scalac` `–d classes HelloWorld.scala`

Compile a Scala program using a user-defined `java` command
:   `env JAVACMD``=/usr/local/bin/cacao` `scalac` `–d classes HelloWorld.scala`

Compile all Scala files found in the source directory `src` to the destination directory `classes`
:   `scalac` `–d classes src/*.scala`

### EXIT STATUS

`scalac` returns a zero exit status if it succeeds to compile the specified input files. Non zero is returned in case of failure.

### AUTHOR

Written by Martin Odersky and other members of the Scala team.

### REPORTING BUGS

Report bugs to `https://github.com/scala/bug/issues`.

### COPYRIGHT

This is open-source software, available to you under the Apache License 2.0. See accompanying "copyright" or "LICENSE" file for copying conditions. There is NO warranty; not even for MERCHANTABILITY or FITNESS FOR A PARTICULAR PURPOSE.

### SEE ALSO

**fsc**(1), **scala**(1), **scaladoc**(1), **scalap**(1)

version 1.0

scalac(1)

March 2012
