# Supplementary material for: Adaptive memory reservation strategy for heavy workloads in the Spark environment
Source: PeerJ Comput Sci. 2024 Nov 13;10:e2460. doi: 10.7717/peerj-cs.2460 (PMC11639302; doi:10.7717/peerj-cs.2460)
Supplement: Supplemental Information 3 [file peerj-cs-10-2460-s003.zip › scala-2.12.11/doc/tools/fsc.html]

xml version="1.1" encoding="iso-8859-1"?


fsc man page


fsc(1)

fsc(1)

USER COMMANDS

### NAME

`fsc` – Fast offline compiler for the Scala 2 language

### SYNOPSIS

`fsc` `[ <options> ] <source files>`

### PARAMETERS

`<options>`
:   Command line options. See **OPTIONS** below.

`<source files>`
:   One or more source files to be compiled (such as `MyClass.scala`).

### OPTIONS

The offline compiler supports all options of `scalac` plus the following:

`–reset`
:   Reset compile server caches.

`–shutdown`
:   Shut down the compilation daemon. The daemon attempts to restart itself as necessary, but sometimes an explicit shutdown is required. A common example is if jars on the class path have changed.

`–server <hostname:portnumber>`
:   Specify compile server host at port number. Usually this option is not needed. Note that the hostname must be for a host that shares the same filesystem.

`–J<flag>`
:   Pass `<flag>` directly to the Java VM for the compilation daemon.

### DESCRIPTION

The `fsc` tool submits Scala compilation jobs to a compilation daemon. The first time it is executed, the daemon is started automatically. On subsequent runs, the same daemon can be reused, thus resulting in a faster compilation. The tool is especially effective when repeatedly compiling with the same class paths, because the compilation daemon can reuse a compiler instance.

The compilation daemon is smart enough to flush its cached compiler when the class path changes. However, if the contents of the class path change, for example due to upgrading a library, then the daemon should be explicitly shut down with `-shutdown`.

Note that the `scala` script runner will also use the offline compiler by default, with the same advantages and caveats.

### EXAMPLE

The following session shows a typical speed up due to using the offline compiler.

```
> fsc -verbose -d /tmp test.scala
\&...
[Port number: 32834]
[Starting new Scala compile server instance]
[Classpath = ...]
[loaded directory path ... in 692ms]
\&...
[parsing test.scala]
\&...
[total in 943ms]

> fsc -verbose -d /tmp test.scala
\&...
[Port number: 32834]
[parsing test.scala]
\&...
[total in 60ms]

> fsc -verbose -d /tmp test.scala
\&...
[Port number: 32834]
[parsing test.scala]
\&...
[total in 42ms]

> fsc -verbose -shutdown
[Scala compile server exited]
```

### ENVIRONMENT

`JAVACMD`
:   Specify the `java` command to be used for running the Scala code. Arguments may be specified as part of the environment variable; spaces, quotation marks, etc., will be passed directly to the shell for expansion.

`JAVA_HOME`
:   Specify JDK/JRE home directory. This directory is used to locate the `java` command unless `JAVACMD` variable set.

`JAVA_OPTS`
:   Specify the options to be passed to the `java` command defined by `JAVACMD`.

    With Java 1.5 (or newer) one may for example configure the memory usage of the JVM as follows: `JAVA_OPTS="-Xmx512M -Xms16M -Xss16M"`

### EXIT STATUS

`fsc` returns a zero exit status if it succeeds to compile the specified input files. Non zero is returned in case of failure.

### AUTHOR

Written by Martin Odersky and other members of the Scala team.

### REPORTING BUGS

Report bugs to `https://github.com/scala/bug/issues`.

### COPYRIGHT

This is open-source software, available to you under the Apache License 2.0. See accompanying "copyright" or "LICENSE" file for copying conditions. There is NO warranty; not even for MERCHANTABILITY or FITNESS FOR A PARTICULAR PURPOSE.

### SEE ALSO

**scala**(1), **scalac**(1), **scaladoc**(1), **scalap**(1)

version 0.5

fsc(1)

March 2012
