# Supplementary figures and images for: Adaptive memory reservation strategy for heavy workloads in the Spark environment
Source: PeerJ Comput Sci. 2024 Nov 13;10:e2460. doi: 10.7717/peerj-cs.2460 (PMC11639302; doi:10.7717/peerj-cs.2460)

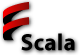

Supplement: Supplemental Information 3 [file peerj-cs-10-2460-s003.zip › scala-2.12.11/doc/tools/images/scala_logo.png]

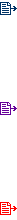

Supplement: Supplemental Information 3 [file peerj-cs-10-2460-s003.zip › scala-2.12.11/doc/tools/images/external.gif]
