# Supplementary material for: Adaptive memory reservation strategy for heavy workloads in the Spark environment
Source: PeerJ Comput Sci. 2024 Nov 13;10:e2460. doi: 10.7717/peerj-cs.2460 (PMC11639302; doi:10.7717/peerj-cs.2460)
Supplement: Supplemental Information 4 [file peerj-cs-10-2460-s004.tgz › spark-2.4.8/docs/_includes/nav-left-wrapper-sql.html]

### Spark SQL Guide

{% include nav-left.html nav=include.nav-sql %}
