# Supplementary material for: Adaptive memory reservation strategy for heavy workloads in the Spark environment
Source: PeerJ Comput Sci. 2024 Nov 13;10:e2460. doi: 10.7717/peerj-cs.2460 (PMC11639302; doi:10.7717/peerj-cs.2460)
Supplement: Supplemental Information 4 [file peerj-cs-10-2460-s004.tgz › spark-2.4.8/docs/_includes/nav-left.html]

{% assign navurl = page.url | remove: 'index.html' %}

{% for item in include.nav %}- {% if navurl contains item.url %}
  **{{ item.text }}**
  {% else %}
  {{ item.text }}
  {% endif %}
{% assign tag = item.url | remove: ".html" %}
{% if item.subitems and navurl contains tag %}
{% include nav-left.html nav=item.subitems %}
{% endif %}
{% endfor %}
