# Supplementary material for: Adaptive memory reservation strategy for heavy workloads in the Spark environment
Source: PeerJ Comput Sci. 2024 Nov 13;10:e2460. doi: 10.7717/peerj-cs.2460 (PMC11639302; doi:10.7717/peerj-cs.2460)
Supplement: Supplemental Information 4 [file peerj-cs-10-2460-s004.tgz › spark-2.4.8/docs/_layouts/404.html]

Page Not Found :(


# Not found :(

Sorry, but the page you were trying to view does not exist.

It looks like this was the result of either:

- a mistyped address
- an out-of-date link
