# Supplementary material for: Adaptive memory reservation strategy for heavy workloads in the Spark environment
Source: PeerJ Comput Sci. 2024 Nov 13;10:e2460. doi: 10.7717/peerj-cs.2460 (PMC11639302; doi:10.7717/peerj-cs.2460)
Supplement: Supplemental Information 4 [file peerj-cs-10-2460-s004.tgz › spark-2.4.8/docs/_layouts/global.html]

  


{{ page.title }} - Spark {{site.SPARK\_VERSION\_SHORT}} Documentation
{% if page.description %}
{% endif %}
{% if page.redirect %}

{% endif %}


{% production %}
{% endproduction %}


{{site.SPARK\_VERSION\_SHORT}}

- Overview
- Programming Guides
  - Quick Start
  - RDDs, Accumulators, Broadcasts Vars
  - SQL, DataFrames, and Datasets
  - Structured Streaming
  - Spark Streaming (DStreams)
  - MLlib (Machine Learning)
  - GraphX (Graph Processing)
  - SparkR (R on Spark)
- API Docs
  - Scala
  - Java
  - Python
  - R
  - SQL, Built-in Functions
- Deploying
  - Overview
  - Submitting Applications
  - Spark Standalone
  - Mesos
  - YARN
  - Kubernetes
- More
  - Configuration
  - Monitoring
  - Tuning Guide
  - Job Scheduling
  - Security
  - Hardware Provisioning
  - Building Spark
  - Contributing to Spark
  - Third Party Projects

{% if page.url contains "/ml" or page.url contains "/sql" %}
{% if page.url contains "/ml" %}
{% include nav-left-wrapper-ml.html nav-mllib=site.data.menu-mllib nav-ml=site.data.menu-ml %}
{% else %}
{% include nav-left-wrapper-sql.html nav-sql=site.data.menu-sql %}
{% endif %}


{% if page.displayTitle %}

# {{ page.displayTitle }}

{% else %}

# {{ page.title }}

{% endif %}
{{ content }}

{% else %}

{% if page.displayTitle %}

# {{ page.displayTitle }}

{% else %}

# {{ page.title }}

{% endif %}
{{ content }}

{% endif %}
