# Supplementary material for: Adaptive memory reservation strategy for heavy workloads in the Spark environment
Source: PeerJ Comput Sci. 2024 Nov 13;10:e2460. doi: 10.7717/peerj-cs.2460 (PMC11639302; doi:10.7717/peerj-cs.2460)
Supplement: Supplemental Information 4 [file peerj-cs-10-2460-s004.tgz › spark-2.4.8/python/docs/_templates/layout.html]

{% extends "!layout.html" %}
{% set script\_files = script\_files + ["\_static/pyspark.js"] %}
{% set css\_files = css\_files + ['\_static/pyspark.css'] %}
{% block rootrellink %}
{{ super() }}
{% endblock %}
